# Traumatic Brain Injury—A Review of Intravenous Fluid Therapy

**DOI:** 10.3389/fvets.2021.643800

**Published:** 2021-07-09

**Authors:** Armi Pigott, Elke Rudloff

**Affiliations:** BluePearl Specialty + Emergency Pet Hospital, Glendale, WI, United States

**Keywords:** traumatic brain injury, TBI, fluid therapies, colloid, crystalloid, osmotherapy, mannitol, hypertonic saline

## Abstract

This manuscript will review intravenous fluid therapy in traumatic brain injury. Both human and animal literature will be included. Basic treatment recommendations will also be discussed.

## Introduction

It is reported that up to 34% of dogs and cats sustaining blunt force trauma will have head and neck injury. In 10% with mild head injury and 80% with severe head injury, intraparenchymal and extra axial hematomas have been detected with advanced imaging (1). Evidence of head trauma is significantly associated with mortality in dogs suffering blunt force trauma (2) and the overall mortality rate is reported to be 24–35% (3, 4). Therapeutic interventions for treating dogs and cats with traumatic brain injury (TBI) are extrapolated from experimental evidence, isolated veterinary reports, human clinical investigations, and anecdotal experience. Confounding injuries, including hemorrhage and additional organ injury, can complicate the decision-making process as well as outcome. Understanding the unique anatomy of the blood brain barrier (BBB) and autoregulation of blood flow, and how they become affected by trauma can provide the clinician with a foundation from which to write a fluid prescription for the patient with TBI.

## Normal Blood Brain Barrier

The brain is highly dependent on a continuous and regulated supply of oxygenated blood traveling through a highly regulated conduit lined by the BBB. The BBB is a physical, transport, and metabolic wall that separates the contents of the blood vessels from the brain interstitium and cells. The endothelial cells lining the vessels of the brain are fenestrated by transmembrane proteins (occludin and claudins or junctional adhesion molecules) anchored to the cytoplasmic surface by scaffolding proteins (zonula occludens) that physically control particle movement through the intercellular clefts and paracellular pathways (5). The capillary membrane is incompletely swathed by pericytes, and together they are encased by a basement membrane constructed by extracellular matrix molecules. Astrocytes extend cellular processes that encase the vessel, neuronal synapses, and nodes of Ranvier, which together make the neurovascular unit. Specific transport mechanisms mediate solute movement across the BBB, and enzymes metabolizing molecules in transit act as a metabolic barrier.

An intact BBB acts as a solute exchange barrier between circulating blood and the brain environment, and functions to allow nutrient delivery and waste removal while limiting entry of immune cells, pathogens, and toxins. The intact BBB is permeable to oxygen, water, and small lipid soluble molecules.

## Blood Brain Barrier Disruption in Traumatic Brain Injury

Dysfunction of the BBB precipitates several key events (6). Paracellular transport of restricted components, in particular neutrophils, increases with the loss of tight junction proteins, and transcytosis of larger molecules such as serum proteins increases across the endothelial cell. This establishes an inflammatory response and an increase in interstitial fluid resulting in vasogenic edema. In addition, activation of cellular membrane ion channels results in intracellular water accumulation and cytotoxic edema, culminating in an increase in brain volume. Following a traumatic event, brain edema will be heterogenous, and alterations in blood flow and oxygen delivery will depend on the severity and region(s) affected.

Since the brain parenchyma is protected within a non-distensible calvarium, an increase in brain volume from edema will increase ICP and reduce CPP in a non-linear manner, resulting in brain ischemia, the single most important secondary insult that can occur following TBI. Therapeutic goals in mitigating the reduction in CPP include optimizing systemic MAP, and, when necessary, decreasing intracranial volume with osmotherapy.

## CPP and Optimizing Map

Blood flow to the normal brain is minimally affected with a MAP between 50–150 mmHg due to autoregulatory mechanisms. A traumatic insult to the brain is followed by disruption of the BBB and cellular injury, and infiltration of inflammatory cells. Their release of cytokines induces nitric oxide production resulting in vasodilation and failure of cerebral pressure autoregulation. Cerebral blood flow in the injured region then becomes dependent on CPP. Systemic hypotension becomes a major contributor to a reduction in CPP, and therefore must be corrected and prevented. Causes of hypotension in patients with TBI can include hemorrhage, third-space fluid losses, and vasoplegia. In addition, polytrauma is common in patients with TBI, and multiorgan damage resulting in hypoxemia, hypovolemia, and systemic inflammation can contribute to the secondary insult to injured brain tissue and complicate the approach to treatment.

Intravenous (IV) fluid therapy is the mainstay of fluid resuscitation from hypovolemia regardless of the extent of trauma. Fluid types include a balanced, buffered, isotonic crystalloid (e.g., Plasma-Lyte, Normosol-R), an isotonic crystalloid with a higher sodium concentration (0.9% sodium chloride), hypertonic saline (HTS 3–7.5%), and/or a synthetic or natural colloid. It can be argued that hyponatremic fluids [e.g., lactated Ringer's solution (LRS)] should be avoided unless the patient is hyponatremic, since they might produce an increased osmolar gap that could favor brain water accumulation (7). A discussion on studies relevant to fluid therapy in animals and people with TBI follows.

## Resuscitation Fluids in Patients With Traumatic Brain Injury and Hemorrhagic Shock

Widely recognized for their guidelines for treating TBI, the Brain Trauma Foundation (BTF) (8) and the Lund Concept (9) have published controversial recommendations for the treatment of TBI. The BTF interventions are based on a set of evidence-based recommendations gleaned from a literature review of published studies, and the BTF guidelines do not make any recommendations about the use of any specific fluid type. The Lund Concept describes non-individualized, pre-emptive, ICP-regulating and perfusion-targeted therapy for manipulating transcapillary fluid dynamics using albumin (in addition to vasodilators and avoiding the use of vasopressors), but lacks strong evidence supporting the protocol (10, 11). There are no clinical trials evaluating any fluid type for resuscitation from hemorrhagic shock in veterinary patients with TBI. There are however dog, cat, pig, rat, and mouse models of hemorrhagic shock and TBI that have been used in the laboratory setting, as well as human clinical trials, attempting to identify the optimal fluid for resuscitation.

### Isotonic Crystalloids

Isotonic crystalloid solutions have been evaluated in the laboratory and human clinical trial setting. They are often the first-line therapy in the pre-hospital environment. However, crystalloids lack any pro-survival properties (12) and there is no survival benefit associated with aggressive crystalloid resuscitation in bleeding patients (13, 14). Modern damage control resuscitation guidelines for hemorrhaging patients recommend avoidance of crystalloid fluids in favor of early initiation of a 1:1:1 ratio-based transfusion strategy using packed red blood cells, plasma, and platelets (15). This strategy may mitigate hemodilution, hemostatic derangements, brain edema, and inflammation associated with large volume crystalloid infusion and worsening of uncontrolled hemorrhage (9, 15–17). In a pig model of TBI and uncontrolled hemorrhage, 100% of pigs died in less than one hour when aggressively resuscitated with isotonic crystalloid solution to a MAP of 80 mmHg, while 50% of pigs that were allowed to remain hypotensive with no resuscitation for one hour survived and went on to have cerebral blood flow return to normal in the second hour following surgical hemostasis and resuscitation with shed blood (18). Hypotensive resuscitation during damage control resuscitation is contraindicated in people with TBI, where resuscitation to a systolic BP of 90–110 mmHg with limited crystalloid infusion is recommended (15).

When compared to synthetic colloids, animals resuscitated with 0.9% sodium chloride or LRS required larger volumes of fluid to reach and maintain hemodynamic endpoints, developed progressive acidosis, and were volume-dependent to maintain MAP and CPP (19, 20). Other animal model studies found that resuscitation from hemorrhagic shock with isotonic crystalloid solutions was associated with lower CPP, higher ICP, lower MAP, higher glutamate-mediated excitotoxic secondary brain injury and increased mitochondrial dysfunction, lower brain tissue oxygenation, more brain edema, larger brain lesion size, upregulation of inflammatory pathway genes, increased activation of coagulation, anticoagulation, and endothelial systems, greater degree of neurologic impairment, and markedly slower rate of neurologic recovery when compared to plasma products, regardless of the type of TBI model studied (17, 19–28).

### Synthetic Colloids

Synthetic colloids, in particular hydroxyethyl starch (HES), are readily available, but their use in critically ill human patients is limited primarily due to increased rates of acute kidney injury and need for renal replacement therapy following administration (29). There are relatively few studies evaluating synthetic colloids in TBI patients, and all but one used experimental animal models. Formation of cerebral edema was greater in a rat model of mild to moderate TBI resuscitated with isovolemic hemodilution using 0.9 or 0.45% sodium chloride compared to whole blood or 6% HES 670/0.75, possibly from a reduction in colloid osmotic pressure (COP) (30). In pig models of TBI and hemorrhage testing various crystalloid and colloid infusion, resuscitation with 6% HES 670/0.75 in LRS required less total volume to achieve hemodynamic endpoints, resulted in a steady improvement in base excess and a CPP >70 mmHg by 270 min post-injury and resuscitation (19, 20). Animals were hypercoagulable in both the LRS and HES groups based on thromboelastographic testing, and there was no difference in transfusion requirement, time to wean from the ventilator, or mortality compared to animals resuscitated with 0.9% sodium chloride. In another pig model of TBI with polytrauma and hemorrhage, 0.9% sodium chloride, 6% HES 670/0.75, and fresh frozen plasma (FFP) were compared as resuscitation fluids (24). HES reduced edema and lesion size compared to 0.9% sodium chloride, but not as effectively as FFP.

The only identified clinical study specifically evaluating synthetic colloids in patients with TBI was a single-center retrospective cohort study of 171 people with severe TBI (31). In this cohort 78% of patients received 6% HES 200/0.5 during hospitalization. There was no association with mortality, change in serum creatinine, or establishment of renal injury. The Crystalloid vs. Hydroxyethyl Starch Trial (CHEST) evaluated 6% HES 130/0.4 and pre-specified a TBI subgroup analysis (32). However, only a small number of patients with TBI were recruited preventing any reliable conclusion (32, 33).

### Natural Colloids

The rationale for using natural colloids as a resuscitation fluid is to avoid or reduce the amount of isotonic crystalloid fluid infused thereby avoiding the complications such as increased brain edema (9, 34), and to avoid use of the synthetic colloids, which, in people, is associated with significant adverse outcomes in many critically ill populations (29). Albumin as a resuscitation fluid during TBI has been evaluated in animal models and human clinical trials with conflicting results. The most notable study was the SAFE (Saline vs. Albumin Fluid Evaluation) trial and subsequent *post-hoc* analysis (34, 35). The SAFE trial was a randomized controlled trial comparing 4% albumin to 0.9% sodium chloride for resuscitation from hemorrhagic shock. A secondary analysis of the subset of patients with hemorrhagic shock and TBI found the patients resuscitated with 4% albumin had higher mortality rate than the subset resuscitated with 0.9% saline (34, 36). This was in contrast to smaller single center and animal studies that suggested a beneficial effect of albumin (37, 38). The mechanism for this outcome cannot be determined from the SAFE trial because the study was not designed to answer this question (9, 34, 36).

Resuscitation with hypoosmolar solutions (including 4% albumin) has been associated with increased brain edema (36). These authors postulate that this may be the reason for increased mortality associated with 4% albumin resuscitation, and that the osmolality of an infusion solution rather than the COP may be more important in the pathogenesis of cerebral edema formation associated with resuscitation fluids (9, 36). Other authors suggest that with a loss of BBB integrity, any colloid might leak into the brain and pull water with it (39). The Lund Concept recommendations continue to support the use of 4% albumin (9) in spite of the evidence of harm (34, 36).

There is increasing evidence in the general trauma population that ratio-based resuscitation with high ratios of FFP to packed red blood cells confers a survival advantage to patients requiring massive transfusion (40). This may be due to avoidance of the complications associated with large-volume crystalloid resuscitation (41, 42). There is also evidence that FFP exerts a protective effect on the endothelium and endothelial glycocalyx layer (22, 41–43), and may protect or help to heal the BBB when administered early to patients with TBI (22, 42, 43). Plasma products have been evaluated as a resuscitation fluid in animal models and human patients with TBI. Fresh frozen plasma, lyophilized plasma, and spray-dried plasma perform similarly when compared to one another (23, 28, 43, 44), and consistently outperform resuscitation with crystalloid or colloid solutions (21–23, 26–28, 36, 42, 44–49).

In animal models of TBI, use of plasma products consistently resulted in favorable responses when compared to resuscitation with isotonic crystalloids (21–23, 25–28, 40–45, 47) or HES (26). Resuscitation from hemorrhagic shock with plasma products was associated with improved CPP, higher MAP, improved brain tissue oxygenation, and reduced brain edema and lesion size (21–23, 26). In addition, administration of plasma products resulted in diminished glutamate-mediated excitotoxic secondary brain injury and reduced mitochondrial dysfunction (21, 26), down-regulation of inflammatory pathway genes and expression of gene clusters mapping to increased metabolic and platelet signaling (26), a lesser degree of neurologic impairment, and markedly faster rate of neurologic recovery (28).

The results of human clinical data surrounding the use of plasma products to treat patients with TBI are mixed and complicated by small sample size and differences in protocols and study population. There are two single center, prospective, randomized trials evaluating the early empirical use of FFP in patients with severe closed head injury: one with 63 patients receiving 5 ml/kg (50) and one with 90 patients receiving 10–15 ml/kg (51). Fresh frozen plasma or an equal volume of 0.9% sodium chloride was administered over 3–4 h following initial hemodynamic stabilization and CT scan. The fluid types and volumes used for initial stabilization are poorly described but may have included blood products, crystalloids, and/or colloids. In both studies, early empirical use of FFP was associated with an increase in delayed traumatic intracerebral hematoma formation. The study by Zhang (50) showed increased rate of blood transfusion and coagulopathy, but no mortality difference in patients receiving FFP, whereas the study by Etemadrezaie (51) showed increased mortality but no difference between groups for rate of coagulopathy (there was no comment on transfusion requirements).

Gruen (46) et al. reported the secondary analysis of a predefined subgroup of patients with TBI from the PAMPer trial. The PAMPer trial (52) was a multi-center, cluster-randomized, phase-3 superiority clinical trial comparing plasma administration to standard-care resuscitation in severely injured patients during air-medical transport, and the primary outcome was mortality at 30 days. The study enrolled patients transported from an outside referral emergency department and directly from the scene of the accident. Patients were randomized to receive plasma vs. no plasma in addition to standard care. From that cohort, a subset of patients with TBI were included in the secondary analysis. Among patients with TBI, the group receiving resuscitation with plasma during air transport had improved 30-day survival compared to those that did not. They also received less crystalloid fluid, vasopressors, and packed red blood cells in the first 24 h, had lower international normalized ratios, lower 24 h mortality, and lower 30-day mortality. The plasma group also had higher incidence of multiple organ failure, longer ICU stay, and longer hospital length of stay. Plasma treatment was associated with the greatest survival benefit in the sickest/most severely injured of these patients. Additionally, transport origin (scene of accident vs. hospital transfer) was used as a proxy for time-to-plasma resuscitation. When grouped by transport origin, patients transported from the scene of the accident who received plasma had lower 30-day mortality than those who did not receive plasma, while there was no difference between patients receiving plasma vs. no plasma when transported from a referral emergency department, suggesting that minimizing time from injury to administration may be important (46).

Retrospective studies also suggest patients with TBI benefit from early resuscitation with plasma (40, 53). Unlike the two single-center prospective trials already discussed, patients in these retrospective trials received plasma as part of the initial resuscitation. Jokar (53) et al. report on 1:1:1 (plasma:pRBC:platelet) ratio-based-resuscitation vs. non-ratio-based resuscitation in trauma patients with isolated TBI and intracranial hemorrhage. Patients receiving ratio-based resuscitation received more plasma and no crystalloid compared to non-ratio-based resuscitation, had significantly lower mortality compared to those who did not, and crystalloid administration was associated with increased odds of death. Additionally, there was no difference in progression of intracranial hemorrhage or rate of neurosurgical intervention between groups (53).

Chang (39) et al. evaluated early plasma transfusion during initial resuscitation in patients with isolated TBI without polytrauma and intracranial hemorrhage at a single center. Evaluation of the full cohort showed no difference in baseline characteristics or survival between patients receiving plasma and those who did not. Patients were then sub-grouped based on the dominant brain lesion: extradural hematoma, subdural hematoma, intraparenchymal contusion, subarachnoid hemorrhage, or multifocal intracranial hemorrhage. There were significant differences in age, mechanism of injury, hypoperfusion, injury severity, early plasma transfusion, and survival among the different subgroups. In the subgroup with multifocal intracranial hemorrhage early plasma transfusion was associated with improved survival. Compared to patients with extradural hematoma, subdural hematoma, intraparenchymal contusion, or subarachnoid hemorrhage, these patients were more likely to present with markers of more severe injury: severe TBI, hypotension, hypoperfusion, more severe injuries, and coagulopathy. Twenty-five percent of these patients received early plasma transfusion which was associated with improved in-hospital survival. Early plasma transfusion was not associated with improved survival in any of the other subgroups (39). It is difficult to make direct comparisons between any of these studies due to the significant differences in populations, protocols, and study design.

### Hyperosmolar Fluids During Resuscitation

An intact BBB is required for a predictable response to osmolar gaps to occur (54–56). Following a significant TBI, cerebral edema may be reduced by a hyperosmolar fluid infusion (54, 57–59). Use of hyperosmolar solutions during initial resuscitation is not discussed in either of the human guidelines (8, 9) although they are evaluated in the experimental and human clinical literature. When used empirically in the prehospital setting as a low-volume resuscitation fluid, 7.5% HTS is well-tolerated but does not confer a survival benefit compared to isotonic crystalloid resuscitation (33, 60, 61). However, in patients with intracranial hypertension both HTS (3–23.4%) and mannitol effectively lower ICP (33, 62). In two experimental dog models of TBI and hemorrhage the animals were resuscitated with either 3% HTS (8 mL/kg) or LRS (16 mL/kg) (63, 64). Animals resuscitated with HTS had higher CPP, lower ICP, higher serum sodium and osmolarity, less cerebral edema, and faster return of pupil responses compared to animals resuscitated with LRS. When animals were further resuscitated by returning their shed blood to maintain MAP >70 mmHg there was no difference in total volume infused between groups (64). In a rat model of TBI and hemorrhage, HTS (7.5%) resuscitation was associated with improved long-term neuronal survival as well as faster and more complete behavioral recovery compared to 0.9% sodium chloride or no resuscitation (65).

## Fluid Resuscitation Technique

The fluid administration and shock management technique might also matter, particularly in patients with uncontrolled hemorrhage. Current BTF (8) and damage control resuscitation guidelines (15) recommend strongly against hypotensive resuscitation in favor of resuscitation to a systolic BP 90–110 mmHg. There is evidence that even transient episodes of hypotension lead to irreversible secondary brain damage in a time- and dose-dependent manner (66–68). However, experimental data suggests normotensive resuscitation prior to hemorrhage control may not be the optimal strategy. Vrettos (18) et al. compared aggressive crystalloid resuscitation to no initial resuscitation in a pig model of TBI with hemorrhage. TBI was induced in anesthetized pigs followed by abdominal hemorrhage to a MAP of 30 mmHg. After 6 min of hypotension the animals were randomized and either resuscitated to a systolic BP of 80 mmHg with LRS or allowed to remain hypotensive. Animals surviving to 1 h post-injury then underwent surgical hemostasis and 1 h of resuscitation with shed blood. All animals in the early aggressive fluid resuscitation group died of exsanguination and hemorrhagic shock in less than an hour, none surviving to undergo surgical hemostasis and further resuscitation. Half of the animals in the hypotensive group survived to receive surgery and resuscitation. In the survivors, MAP, cardiac output, cerebral blood flow and oxygen measurements were restored to pre-hemorrhage levels. There was no evaluation of brain lesion size or any functional outcome in these animals. The authors suggest that while hypotension is suboptimal in TBI, bleeding to death leaves no chance of survival, and other resuscitation strategies need to be investigated.

In another pig TBI and uncontrolled abdominal hemorrhage model, resuscitating pigs with vasopressin plus 6% HES 670/0.75 in LRS increased blood pressure but failed to improve cerebral blood flow and increased abdominal hemorrhage volume compared to resuscitation with HES only and to no resuscitation (66). A third pig TBI and hemorrhage model compared FFP and 0.9% sodium chloride administered as either a large rapid bolus or slower stepwise resuscitation (27). Pigs underwent TBI and 40% blood loss, were kept hypotensive for 2 h, then resuscitated with FFP or 0.9% sodium chloride. The FFP group received the shed blood volume back as either a fast bolus (50 ml/min) or stepwise infusion starting at 2 mL/min and gradually increasing to 50 mL/min. The 0.9% sodium chloride group received 3x the shed blood volume either as a bolus at 165 mL/min or starting at 6 ml/min and gradually increasing to 165 mL/min. Animals were euthanized and tissue harvested after 6 h. Bolus FFP or 0.9% sodium chloride resulted in greater brain swelling but similar lesion size to stepwise FFP or 0.9% sodium chloride, suggesting that stepwise infusion is superior to rapid bolus. In addition, 0.9% sodium chloride infusion resulted in more swelling and a larger brain lesion when compared to both FFP infusion types.

## Managing Elevated ICP With Osmotherapy

Osmotherapy is the infusion of a hyperosmolar fluid with the intention of producing an osmolar gap and transferring brain parenchymal fluid into the vessels to be excreted in the urine. This reduces blood viscosity, which improves rheology resulting in constriction of pial arterioles (68–70). A direct effect at the site of injury may not be realized should blood flow to the site of injury be limited, or the BBB be disrupted. However, in regions where the BBB is intact, the osmolar gap may remove water that has accumulated in the brain cell and interstitium, and reduce ICP. The most common fluids used for osmotherapy include mannitol (20 and 25%) and HTS (3, 7.5, 24%). Their differences are summarized in Table 1. A key difference between the two fluids is that HTS can be used for dual purpose in treating hypovolemic shock as well as reduce cerebral edema. There is no strong evidence to support any recommendation for the use of osmotherapy for the treatment of traumatic intracranial hypertension or using one over another, and a summary of the evidence follows.

**Table 1:** Characteristics of Mannitol and Hypertonic Saline.

	**Mannitol**	**Hypertonic Saline**
Base	6-carbon, alcohol sugar isomer of sorbitol	Sodium and chloride
Osmolarity (mOsm/L)	20%: 1100 25%: 1375	3%: 1027 (500 mmol/L)7.5%: 2565 (1274 mmol/L)23.4%: 8008 (4004 mmol/L)
Reflection coefficient	0.9	1.0
Molecular weight (Daltons)	182	
Diuretic effect	Excreted as is in urine and induces osmotic diuresis	Releases ANP
Additional effects	Oxygen-free radical scavenger	↑IV volume & ↑MAP Immunomodulator
Dose for treating intracranial hypertension	500–1000 mg/kg over 15 min. Can be repeated 2-3 X if effective	4-6 ml/kg of 7.5%
Dose for treating hypovolemia		4-7 ml/kg of 7.5% or 4-7 ml/kg 1 part 23.4% mixed with 3 parts HES

A single clinical veterinary study evaluating the effect of isosmotic mannitol and 3% HTS in two cats and one dog was identified (71). The animals were presented to a veterinary teaching hospital with naturally occurring head trauma, received immediate cardiovascular resuscitation with LRS, pain control with fentanyl, and antibiotic coverage with cefazolin when indicated. A brain MRI was performed within 12 h of presentation and as soon as the animals were considered stable. Animals suspected to have elevated ICP were instrumented for direct ICP monitoring immediately following imaging and randomized to receive either 18% mannitol or 3% HTS. ICP and CPP were recorded before and at five timepoints during the 120 min post-treatment. Patient one received 3% HTS and had no response to treatment. Patient two received 3% HTS resulting in an approximately 40% decrease in ICP and 15% increase in CPP. The ICP remained lower than baseline however the patient became hypotensive requiring further isotonic fluid resuscitation and dopamine to raise the MAP. This period of hypotension resulted in a decreased CPP, and the patient's response to 3% HTS was therefore classified as transient. The third patient received 18% mannitol. Initially the ICP decreased by 19% and the CPP returned to normal, however there was a rebound increase in ICP that was higher than pre-treatment values, and the CPP decreased again before gradually returning to normal over the 120-min monitoring period.

Numerous reviews and meta-analyses evaluate HTS and mannitol against various agents in the human clinical literature (33, 62, 72–82). In summary, the studies are heterogenous in population, dose, concentration and rate of fluid administered, therapeutic targets, and outcomes of interest. Therefore, the systematic reviews can only draw limited and general conclusions. The available evidence suggests that both mannitol and HTS effectively lower ICP, but there is not enough evidence to suggest one fluid is superior, although a few studies suggest HTS may have slightly fewer treatment failures in patients with refractory intracranial hypertension compared to mannitol (62, 73–76, 78, 83, 84). Additionally, HTS avoids diuresis and increases cardiac preload, favorably impacting cerebral perfusion (33, 79). Mannitol is associated with a well-documented rebound phenomenon in patients with intracranial hemorrhage and brain tumors, occurring in about 12% of patients (85–89). A rebound phenomenon was defined in a Cochrane review by Chen (76) et al. as ‘intracranial pressure rising above its original level after hyperosmolar therapy.' However, the rebound phenomenon occurring in patients with TBI is only mentioned in passing in a single study included in the Cochrane review (76) and in the veterinary pilot study (71). Despite effective reduction of ICP, neither HTS nor mannitol has clinical evidence supporting improved survival or long-term neurologic outcome (33, 75, 81).

The optimal dose has not been determined for either mannitol or HTS. There is some evidence that higher doses of mannitol (~ 1.0–1.5g/kg) might be associated with greater reduction of ICP and less rebound phenomenon compared to lower doses of mannitol, although the data surrounding the dose-relationship with rebound phenomenon is conflicting (80, 90–93). A specific, evidence-based dosing strategy for HTS cannot be determined at this time due to insufficient evidence and profound heterogeneity among the various studies. In the veterinary clinical study (71) noted previously, the 2 cats received 5.3 ml/kg of 3% HTS IV over 5 min. One had no response to therapy and the other had an approximately 40% decrease in ICP. The Neurocritical Care Society has published guidelines for acute treatment of cerebral edema in human neurocritical care patients with TBI and recommends symptom-based bolus dosing over sodium-target-based dosing (94).

Continuous infusion of HTS in patients with various pathologies has been evaluated in a small single center trial and was compared in a pooled analysis against intermittent bolus therapy from two other trials (95). The hazards ratio for survival showed a 90-day functional outcome with continuous infusion to be significantly greater compared to bolus therapy. There were no significant adverse effects observed with HTS continuous infusion (96, 97). This is in contrast to pediatric patients, where sustained hypernatremia is associated with thrombocytopenia, kidney failure, neutropenia, and ARDS (97–101). The recently published COBI (Continuous hyperosmolar therapy (20% HTS) in Brain-Injured patients) trial compared functional outcome at 6 months between standard care alone and continuous therapy with 20% HTS in 370 adults (102). All patients received recommended interventions based on the most recent BTF guidelines. The treatment group received a 1 h bolus infusion adjusted to their measured serum sodium level within 24 h of trauma, which was immediately followed by a 0.5–1 g/kg/h continuous infusion of saline. Serum sodium levels were monitored, and the infusion concentration adjusted to prevent an elevation in serum sodium >155 mmol/L for a minimum treatment period of 48 h and only while intracranial hypertension remained a risk. The infusion was stopped once 12 h had passed following the suspension of all specific therapies for intracranial hypertension. Although the study was underpowered to detect a clinically important difference, the authors concluded that in patients with moderate to severe TBI, there was no significant difference in neurological status between the treatment and control group.

## Additional Considerations With HTS and Mannitol

Several local and systemic pathophysiological consequences contribute to secondary injury of the brain which may be mitigated by HTS and/or mannitol. Hypovolemia, hypotension, cerebral vasospasm, and altered blood flow result in activation of systemic inflammation and hypoxemia. In the brain, cerebral leukocytes congregate in injured areas and initiate vasodilation and peroxidase/protease-mediated cell death (101, 103). Dysfunction of cell-mediated immunity can occur and may be moderated by HTS (104–107).

Hypoxemia results in the depletion of ATP, cellular membrane ion pump malfunction, intracellular sodium accumulation and endothelial cell swelling. This can narrow the vascular lumen causing red blood cells to pass through vessels with more difficulty, and rupture or cause premature apoptosis of neuronal cells. In addition, brain injury can induce extensive neuronal depolarization which decreases extracellular sodium reversing the direction of the Na-glutamate cotransporter, causing an increase in extracellular glutamate, compounding neurotoxicity (107–110). Using HTS during resuscitation improves alveolar gas exchange by reducing extravascular lung volume, reverses endothelial and red blood cell swelling improving blood flow and oxygen delivery and restores extracellular sodium and cellular action potential, moderating glutamate toxicity in the brain (111–116).

During reperfusion of hypoxemic tissue, the production of radical oxygen species can propagate tissue injury. Mannitol may limit the secondary oxidative injury in the brain by scavenging radical oxygen species (117).

Hypotension caused by a decrease in systemic vascular resistance and/or a vagal-mediated reflex after the rapid administration of HTS has been reported to occur in humans, dogs, and rabbits (118–120). This appears to be transient as it is followed by an increase in MAP and myocardial contractility.

## Suggested Therapeutic Approach

**Figure 1:**
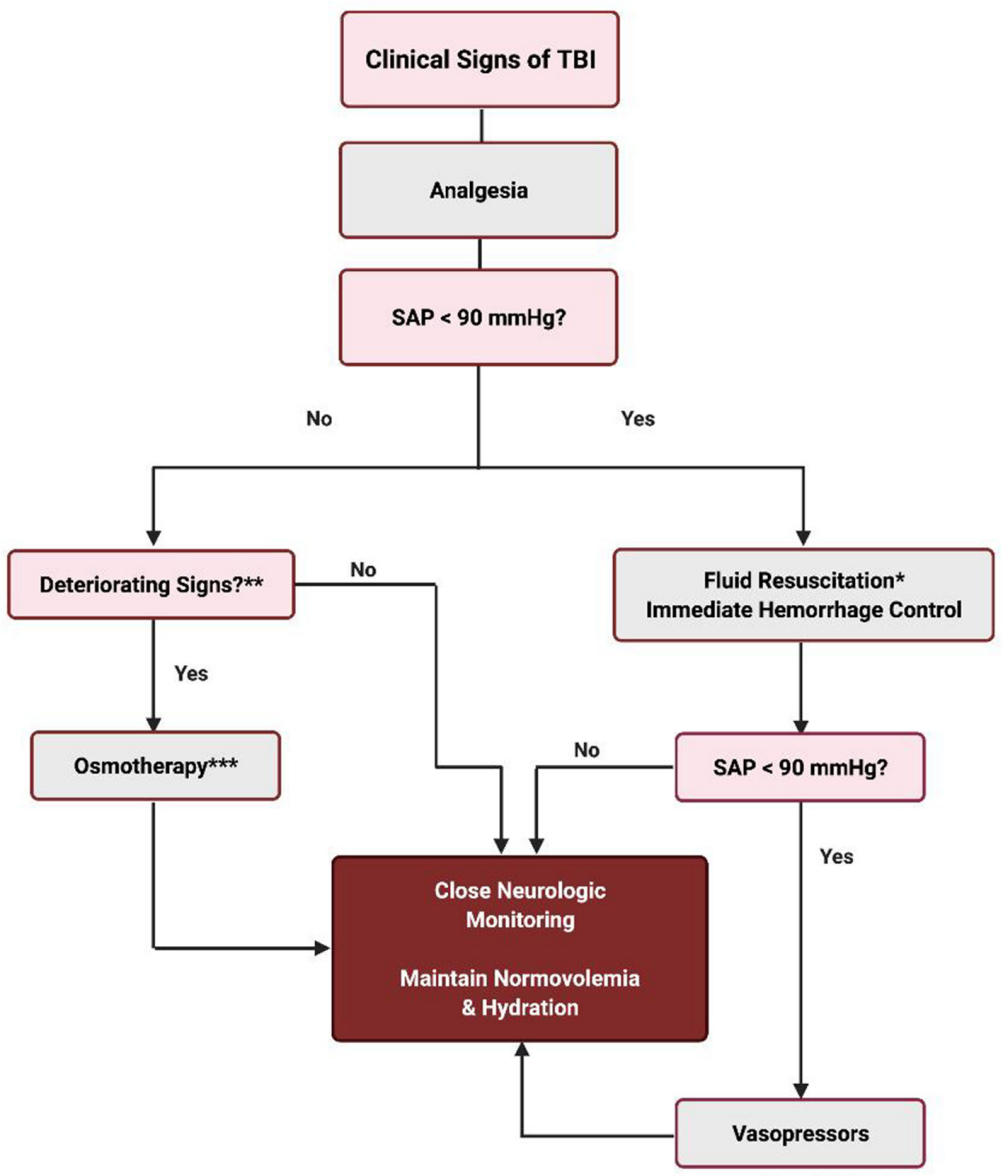
Fluid therapy for the TBI patient. *Fluid resuscitation techniques can be any one of the following or a combination thereof: (1) 10–20 ml/kg crystalloids (Plasma Lyte or Normosol-R) IV rapid infusion up to 60–90 ml/kg. (2) 5–10 ml/kg 6% HES (tetrastarch) IV rapid infusion up to 40–50 ml/kg. (3) 5–10 ml/kg plasma rapid infusion IV up to 20–30 ml/kg. (4) 3–4 ml/kg 7% HTS IV over 10–15 min. (5) whole blood or pRBC, if indicated. **Altered level of consciousness with or without bilateral or unilateral miotic pupils; unresponsive mid range pupil(s) or mydriasis; loss of the oculocephalic reflex; bradycardia with hypertension (Cushing reflex); posturing (opisthotonus, decerebellate, decerebrate); alteration of the respiratory pattern. ***1 g/kg mannitol IV up to 3 doses q60–90 min OR 3–4 ml/kg 7% HTS IV.

It is clear from the information available that therapeutic recommendations for fluid therapy in the dog or cat with TBI continue to remain the clinician's choice. Maximizing CPP by correcting systemic hypotension is a cornerstone to management of TBI, although this has to be done carefully when also treating severe hemorrhage resulting from polytrauma. Hypovolemia is treated with isotonic crystalloids, hypertonic saline, and/or colloids. A decline in neurological status in the non-hypotensive patient warrants osmotherapy. The authors approach to fluid resuscitation of the small animal patient with TBI is outlined in Figure 1.

## On the Horizon

Various fluid additives and novel molecules are being investigated to identify the optimal resuscitation fluid for patients with TBI. One combination that stands out is HTS containing adenosine, lidocaine, and magnesium. This combination appears to play a protective role in a variety of life-threatening conditions in animal models of sepsis (121–124), non-compressible hemorrhagic shock (125–127), and TBI from non-compressible hemorrhage (128). Interestingly, the drugs do not confer benefit when used individually, and magnesium sulfate alone might increase mortality in humans with TBI (129). LRS with added drag-reducing molecules has been evaluated in a rat model, and appears to improve cerebral microcirculation, increase brain tissue oxygenation, and reduce neuron loss, despite lower mean arterial pressure (130).

Modified hemoglobin-based oxygen carriers (HBOCs) that have reduced nitric oxide scavenging and oxygen-free radical generation are being evaluated as resuscitation fluids in animal models of TBI and hemorrhagic shock (131–133). Polynitroxylated-pegylated hemoglobin (PNPH) is a novel HBOC bovine-origin carboxyhemoglobin with covalently labeled nitroxide moieties being evaluated for use as a small-volume resuscitation fluid (132–134). The polynitroxylation of the hemoglobin molecule has antioxidant properties and prevents nitric oxide scavenging, while the polyethylene glycol side chains create a “hydrating shell” that has a strong oncotic effect useful in resuscitation (131). PNPH has also been evaluated in mouse models of TBI with hemorrhage (131–133), and compared with crystalloid and whole blood resuscitation (131). Mice resuscitated with PNPH required smaller volume fluid resuscitation and had higher mean arterial blood pressure that remained normal and stable through to the end of the experiment without the need for additional fluid infusion. In addition, they had a lower ICP and markedly less brain edema compared to those resuscitated with crystalloid or whole blood.

Dodecafluoropentane emulsion is an oxygen-carrying perfluorocarbon emulsion also under investigation for use in patients with TBI. In humans it has a short half-life (90 min) and is cleared via exhalation from the lungs (135). It is administered IV, travels to the lungs where it picks up oxygen, then to the tissues where it delivers oxygen. It has been evaluated in a rat model of TBI where brain tissue oxygen levels increased to 146% of the post-injury, pre-treatment level, with no effect on systemic blood pressure, heart rate, biochemical parameters, or blood gas measurements (135, 136). Further evaluation is needed before these therapies can be recommended in clinical practice.

## Conclusion

There is a paucity of information covering treatment of TBI in dogs and cats, which is limited to experimental data in primarily pigs and rats, and clinical data collected from human studies. Response to treatment can be complicated by acute hemorrhage. The research and limited clinical studies examined do not provide sufficient evidence for a preferred fluid type, although it appears that infusion of LRS is less desirable than other isotonic crystalloids, and the use of plasma products during resuscitation may convey an improved outcome. To further the knowledge base on therapeutic interventions for TBI in dogs and cats, future clinical studies should focus on the effect of specific fluid prescriptions and osmotic agents on short- and long-term outcome.

## Author Contributions

Both authors contributed to the body of the paper, reference research, and editing.

## Conflict of Interest

The authors declare that the research was conducted in the absence of any commercial or financial relationships that could be construed as a potential conflict of interest.
